# GPU-Accelerated Discovery of Pathogen-Derived Molecular Mimics of a T-Cell Insulin Epitope

**DOI:** 10.3389/fimmu.2020.00296

**Published:** 2020-02-28

**Authors:** Thomas Whalley, Garry Dolton, Paul E. Brown, Aaron Wall, Linda Wooldridge, Hugo van den Berg, Anna Fuller, Jade R. Hopkins, Michael D. Crowther, Meriem Attaf, Robin R. Knight, David K. Cole, Mark Peakman, Andrew K. Sewell, Barbara Szomolay

**Affiliations:** ^1^Cardiff University School of Medicine, Cardiff, United Kingdom; ^2^Systems Immunity Research Institute, Cardiff University, Cardiff, United Kingdom; ^3^Zeeman Institute for Systems Biology and Infectious Disease Epidemiology Research, University of Warwick Coventry, Coventry, United Kingdom; ^4^Faculty of Health Sciences, University of Bristol, Bristol, United Kingdom; ^5^Mathematics Institute, University of Warwick, Coventry, United Kingdom; ^6^Peter Gorer Department of Immunobiology, Guy's Hospital, London, United Kingdom

**Keywords:** type 1 diabetes, T-cell receptor, peptide-HLA, insulin, molecular mimicry, general-purpose computing on graphics processing units (GP-GPU), Compute Unified Device Architecture (CUDA), Nvidia

## Abstract

The strong links between (Human Leukocyte Antigen) HLA, infection and autoimmunity combine to implicate T-cells as primary triggers of autoimmune disease (AD). T-cell crossreactivity between microbially-derived peptides and self-peptides has been shown to break tolerance and trigger AD in experimental animal models. Detailed examination of the potential for T-cell crossreactivity to trigger human AD will require means of predicting which peptides might be recognised by autoimmune T-cell receptors (TCRs). Recent developments in high throughput sequencing and bioinformatics mean that it is now possible to link individual TCRs to specific pathologies for the first time. Deconvolution of TCR function requires knowledge of TCR specificity. Positional Scanning Combinatorial Peptide Libraries (PS-CPLs) can be used to predict HLA-restriction and define antigenic peptides derived from self and pathogen proteins. *In silico* search of the known terrestrial proteome with a prediction algorithm that ranks potential antigens in order of recognition likelihood requires complex, large-scale computations over several days that are infeasible on a personal computer. We decreased the time required for peptide searching to under 30 min using multiple blocks on graphics processing units (GPUs). This time-efficient, cost-effective hardware accelerator was used to screen bacterial and fungal human pathogens for peptide sequences predicted to activate a T-cell clone, InsB4, that was isolated from a patient with type 1 diabetes and recognised the insulin B-derived epitope HLVEALYLV in the context of disease-risk allele HLA A*0201. InsB4 was shown to kill HLA A*0201^+^ human insulin producing β-cells demonstrating that T-cells with this specificity might contribute to disease. The GPU-accelerated algorithm and multispecies pathogen proteomic databases were validated to discover pathogen-derived peptide sequences that acted as super-agonists for the InsB4 T-cell clone. Peptide-MHC tetramer binding and surface plasmon resonance were used to confirm that the InsB4 TCR bound to the highest-ranked peptide agonists derived from infectious bacteria and fungi. Adoption of GPU-accelerated prediction of T-cell agonists has the capacity to revolutionise our understanding of AD by identifying potential targets for autoimmune T-cells. This approach has further potential for dissecting T-cell responses to infectious disease and cancer.

## Introduction

T-cells protect against infections and neoplasms by scanning the host proteome for anomalies via peptides bound in major histocompatibility (MHC) molecules at the cell surface. The peptide specificity of T-cells is determined by the T-cell receptor (TCR), a highly variable heterodimeric protein generated by the somatic rearrangement of variable, joining and diversity gene segments and the quasi-random introduction/deletion of nucleotides to bestow additional non-germline-derived variation. Although it is estimated that this process can theoretically generate ~10^18^ different αβ TCRs in human (1) the actual repertoire possessed by any individual is believed to be of the order of 10^8^ distinct TCR sequences (2, 3). The major variation between different TCRs is manifest in the antigen binding site, composed of six composite complementarity-determining region (CDR) loops, which contact both the peptide and the restricting MHC (4, 5). Effective immune coverage requires that the TCR repertoire must be capable of responding to almost all foreign peptide sequences that could be presented by host MHC molecules (1, 6). Unlike the B-cell receptor, the TCR is fixed and is not susceptible to further alteration in amino acid sequence or affinity maturation. There is no time to manufacture new TCRs in the face of an infection, so it is essential that the receptors on peripheral naïve T-cells are capable of dealing with any new challenge. Failure to cover all possible foreign peptide antigens would leave blind spots in T-cell immunity that pathogens could quickly evolve to exploit (1). Thus, successful immunity requires that a repertoire of ~10^8^ TCRs be capable of responding to a vastly greater number (>10^16^) of potential foreign peptides of a length and sequence capable of being presented by self MHC molecules (6). This evolutionary challenge is overcome by a phenomenon termed TCR degeneracy which refers to the ability of individual TCRs to transmit activation signals from very large numbers of different peptides bound in a single MHC molecule (7). The resulting T-cell crossreactivity, where a single T-cell clonotype can respond to a wide array of different peptide sequences, is thus an essential feature of T-cell immunity (1, 6).

Detailed examination of the potential for T-cell crossreactivity to trigger autoimmune disease (AD) will require means of predicting which peptides might be recognised by autoimmune TCRs. Here, we develop tools for this purpose that enabled the complex calculations involved in screening a wider pathoproteome to be undertaken by individual researchers on their own desktop computers. For this purpose, we focused on the AD type 1 diabetes (T1D) which arises through immune destruction of insulin-producing β-cells in the pancreas. T1D is triggered by the direct destruction of insulin-producing cells by cytotoxic T-cells. Four MHC class I molecules, human leukocyte antigen (HLA)*0201 (HLA A2 hereon), HLA A*24, HLA B*18, and HLA B*3906 are risk factors for T1D (8). Post mortem studies of patients close to disease onset have showed that numerous CD8 T-cells present in the characteristic islet mononuclear cell infiltrate (9). Efficient adoptive transfer of disease in animal models typically requires CD8 T-cells and disease in such models is prevented by genetic modifications that abrogate MHC class I expression (10). Transgenic introduction of the HLA allele *HLA A2* into diabetes-prone non-obese diabetic (NOD) mice markedly accelerates disease development (11). More recent studies have described TCRs on activated CD8 T-cells in patient blood that can recognise β-cell-specific peptides presented through known disease risk HLA class I alleles (12–14) and such cells have been placed at the “scene of the crime” by *in situ* tetramer staining of insulitic lesions (15). We previously searched for potential pathogen-derived peptide ligands for the 1E6 TCR which was isolated from a type 1 diabetic and shown to exhibit glucose-mediated killing of human HLA A2^+^ pancreatic beta cells (12, 14, 16). Two agonist peptides identified were mapped to the human pathogens *Bacteroides fragilis/thetaiotaomicron* and *Clostridium asparagiforme* demonstrating the potential utility of this approach for discovery of molecular mimicry (16, 17). These peptide agonists of sequence MVWGPDPLYV and RQFGPDWIVA, respectively, differed from the wildtype insulin-derived sequence, ALWGPDPAAA presented at the cell surface of HLA A2^+^ human beta cells, and from each other, at seven of ten positions highlighting the potential extent of T-cell crossreactivity in this system. Pathogen-derived agonist peptides were identified using positional scanning combinatorial peptide library (PS-CPL) data to search the human pathoproteome (16).

Proteome database searches, in particular those derived from bacterial genomes, are computationally demanding on Central Processing Units (CPUs) and therefore of limited applicability. Here we sought to overcome this limitation using General-Purpose Graphics Processing Units (GP-GPUs) to reduce the execution time by a considerable degree. The great advantage of a using a GP-GPU resides in the ability to parallelise the task at a massive scale, which allows for a huge increase in the number of sub-processes carried out per second (the throughput); even if the total time required to complete a sub-process (the latency) is somewhat increased, the net gains can be considerable. Compute Unified Device Architecture (CUDA), Nvidia's® GPU computing platform, is a free software that allows speed up of compute-intensive applications by harnessing the power of GPUs for the parallelisable part of the computation. CUDA has already been implemented in several bioinformatics tools ranging from molecular dynamics, sequence alignment, structure prediction, and spatio-temporal modelling (18–21). Many of these studies used consumer graphics cards aimed principally at gamers and costing no more than a few hundred pounds. CUDA provides a powerful and cost-effective way of accelerating computations that can be run on desktop setups without the need to access a high-performance computing (HPC) server (22). Use of CUDA and standard desktop computation means source code can be shared among researchers and run independently, allowing for modifications and greater ease of use; it also negates increases in wall time that would be attributed to the nature of HPC, including job queueing and hosting.

We previously developed a peptide scoring algorithm that generates a ranked list of self and corresponding pathogenic viral peptides predicted to act as T-cell agonists from PS-CPL data generated with a given T-cell clone (17, 23). Here we extend this platform to include an expanded human pathoproteome by building protein databases from pathogenic bacteria and fungi and by implementing the peptide scoring algorithm into a GPU-accelerated framework which is suitable in view of the fact that our algorithm fits the single instruction on multiple data (SIMD) paradigm (24).

In summary, we present a GPU-accelerated strategy for epitope discovery and characterisation that uses the CPL-driven peptide scoring method developed by Szomolay et al. (17) to rank bacterial and fungal peptides occurring in very large curated databases in order of likelihood of recognition. Our GPU-implementation of the scoring algorithm significantly reduces the computational time and cost and has the potential to become an efficient application for ligand hunting. C++ is freely downloadable and can be run on any platform. GPU can be also included in most personal computers thereby democratising the approach as a publicly accessible executable, available at https://github.com/WhalleyT/PICPL. The executable contains both the C++ and CUDA-versions of the algorithm. It also contains a test data (a sample of protein sequences and CPL data) that the user can run on any of the three platforms (Windows, Linux, MacOS operating systems) and can be downloaded from the Releases.

## Materials and Methods

### Cell Lines and T-Cells

HLA A2 restricted clone InsB4 that recognises the HLVEALYLV peptide from the insulin chain (residues 10–18) (25) was grown from CD8 T-cells purified from a T1D patient using a T-cell library (26). Clone InsB6 was produced at the same time as InsB4 and subsequently found to express the same TCR. T-cell clones were routinely expanded using irradiated (3,000–3,100 cGy) allogenic PBMCs from three donors and 1 μg/mL of phytohaemaglutinin (PHA) (Alere, Thermo Scientific, Walthan, MA, USA) in T-cell media made from RPMI-1640 supplemented with 10% fetal bovine serum (FBS), 2 mM L glutamine, 100 U/mL penicillin, 100 μg/mL streptomycin, 10 mM HEPES 0.5X non-essential amino acids and 1 mM sodium pyruvate (all from Life Technologies, Carlsband, CA, USA) supplemented with 200 IU of IL-2 (Aldesleukin, Proleukin, Prometheus, San Diego, CA, USA) and 25 ng/mL human IL-15 (Peprotech, Rocky Hill, NJ, USA). T-cells were cultured for 2–4 weeks post-restimulation before being used for assays. K562 cell lines expressing HLA A2 and preproinsulin or GAD65 were generated and cultured as previously described (14, 27). CIRs expressing HLA A2 (28) were grown as suspension cells in R10 medium (RPMI-1640 with 10% FBS, 2 mM L-glutamine, 100 U/mL penicillin and 100 μg/mL streptomycin). Human pancreatic β-cells were produced as previously described (14, 27). T2 cells (HLA A2^+^) were used as peptide presenting cells and cultured as for K562s.

### T-Cell Functional Assays

Functional sensitivity assays using peptides were performed as previously described (29). T-cells were “rested” overnight in R5 (as for R10 but with 5% FBS) to reduce spontaneous release of the chemokine, MIP-1β. Individual peptides were synthesised to >40% purity [GL Biochem (Shanghai) Limited, Shanghai, China] and titrated for incubation overnight in R5 medium with the respective clone. Supernatants were harvested for MIP-1β ELISA using 96 well half area plates and a human MIP-1β DuoSet ELISA kit from R&D Systems (Minneapolis, MN, USA). Cytotoxicity assays using chromium 51 (17) or a non-radioactive europium hydrophilic ligand (14) were performed as described previously, with minor adjustments: for chromium release assays, 2,000 target cells were used per well. Data was initially analysed using GraphPad Prism software to generate the EC_50_ of activation (concentration of peptide at 50% of maximum activation), which was used for further calculations as described below.

### Sizing and Combinatorial Peptide Library (CPL) Scans

While MHC class I-restricted T-cells can recognise vast numbers of different peptides, all the strongest agonists tend to be of a single defined length of between 8 and 14 amino acids (30). The optimal length peptide recognised by InsB4 T-cells was determined by examining the response to six random peptide mixtures, X^8^, X^9^, X^10^, X^11^, X^12^, and X^13^ (where X is any of the 19 proteogenic L-amino acids excluding cysteine) in 96U well-plates in R5 medium (as for R10 but with 5% FBS) as for the functional peptide assays above and described elsewhere (30). MIP-1β was used as a readout as it is the most sensitive CD8 T-cell functional indicator (31, 32). Sizing and CPL scan peptide mixtures were purchased lyophilised at >40% purity (Pepscan Presto BV, Lelystad, The Netherlands) and stored as 80 or 20 mM DMSO stocks respectively, at −80°C. Working stocks (10 or 1 mM, respectively) were prepared in R0 (as for R10 but with no FBS), stored at 4°C, and used in assays at 1 or 0.1 mM, respectively.

### T-Cell Receptor Sequencing and Production of Soluble TCR Protein

The TCRs of InsB4 and InsB6 were sequenced in-house using Sanger sequencing techniques as described previously (33). Individual TCR chains were expressed in *E. coli* as soluble inclusion bodies and refolded as described (34).

### Peptide-MHC Multimer Staining

An optimal staining protocol was used for pMHC multimer staining as previously described (35), involving preincubation with 50 nM of the protein kinase inhibitor Dasatinib (36), followed by staining with 0.5 μg of PE conjugated pMHC tetramer in 50 μL of FACS buffer (2% FBS in PBS), then incubation with an anti-PE unconjugated antibody (23). Subsequently, cells were stained for 5 min at RT with 2 μL of a cell viability stain (Vivid; Life Technologies) that had been diluted 1:40 using PBS then without washing with anti-CD8-APC antibody (clone BW135/80, Miltenyi Biotech, Bergisch Gladbach, Germany). Data acquisition was performed on a BD FACS Canto II (BD Biosciences, Franklin Lakes, NJ, US) and analysed using FlowJo Software (TreeStar, Ashland, OR, US).

### Manufacture of Soluble Proteins

Soluble TCR protein and biotinylated pMHCI were manufactured as previously described (34). Briefly, codon-optimised InsB4 TCR α and β chains, HLA A2 heavy chain and β2m chain were generated by Genewiz. All sequences were confirmed by automated DNA sequencing (Eurofins). TCR expression constructs were designed with a disulphide linked construct to produce the soluble domains (variable and constant) for both the α (residues 1–204) and β chains (residues 1–245) (34). The HLA A2 heavy chain (residues 1–248) (α1, α2, and α3 domains), tagged, or not tagged with a biotinylation sequence, and β2m (residues 1–100) were also cloned and used to make the pMHCI complexes. The TCR α and β chains, the HLA A2 α chain and β2m sequences were inserted into separate pGMT7 expression plasmids under the control of the T7 promoter (5). Competent Rosetta DE3 *E. coli* cells were used to produce the TCR α and β chains, HLA A2 heavy chain and β2m in the form of inclusion bodies (IBs) using 0.5 mM IPTG to induce expression and protein were chemically refolded as described previously (37).

### Surface Plasmon Resonance

SPR Equilibrium binding analysis was performed using a BIAcore T200^TM^ equipped with a CM5 sensor chip as previously reported (38, 39). HLA A2-NLSALGIFST, derived from insulin-like growth factor 2 mRNA binding protein and recognised by a different TCR, was used as a negative control on flow cell 1. SPR kinetic analyses were carried out to determine the K_D_ values for the TCR, at 25°C. For all kinetic experiments, approximately 500 RUs of pMHC was coupled to the CM5 sensor chip surface. The TCR was then injected ten times at serial dilutions, from a concentration of 407 μM, at 30 μl/min. The K_D_ values were calculated assuming 1:1 Langmuir binding [AB = B*AB_MAX_/(K_D_ + B)] and the data were analysed using a global fit algorithm (GraphPad Prism).

### GP-GPU Parallelisation of the Peptide Scoring Algorithm

The code developed by Szomolay et al. (17) was initially written in Matlab because its ease of use enables rapid prototype development and testing. As most of the time was spent scoring amino acid positions in a peptide against a lookup table, and this process could be performed as a matrix operation, we optimised the code and were able to obtain a 10-fold speedup vs. the original prototype algorithm. The code was parallelised via Matlab's Parallel Computing Toolbox and scaled up to multiple hosts in a computational cluster using the Distributed Computing Server, which allows breaking the protein database up into sections and sending each section to a different thread. Results were then collated before the best overall peptide matches were found. The overhead of parallelisation using multithreading in Matlab is not significant and accordingly, the speed gain is virtually linear with respect to the number of parallel threads.

**Figure 1:**
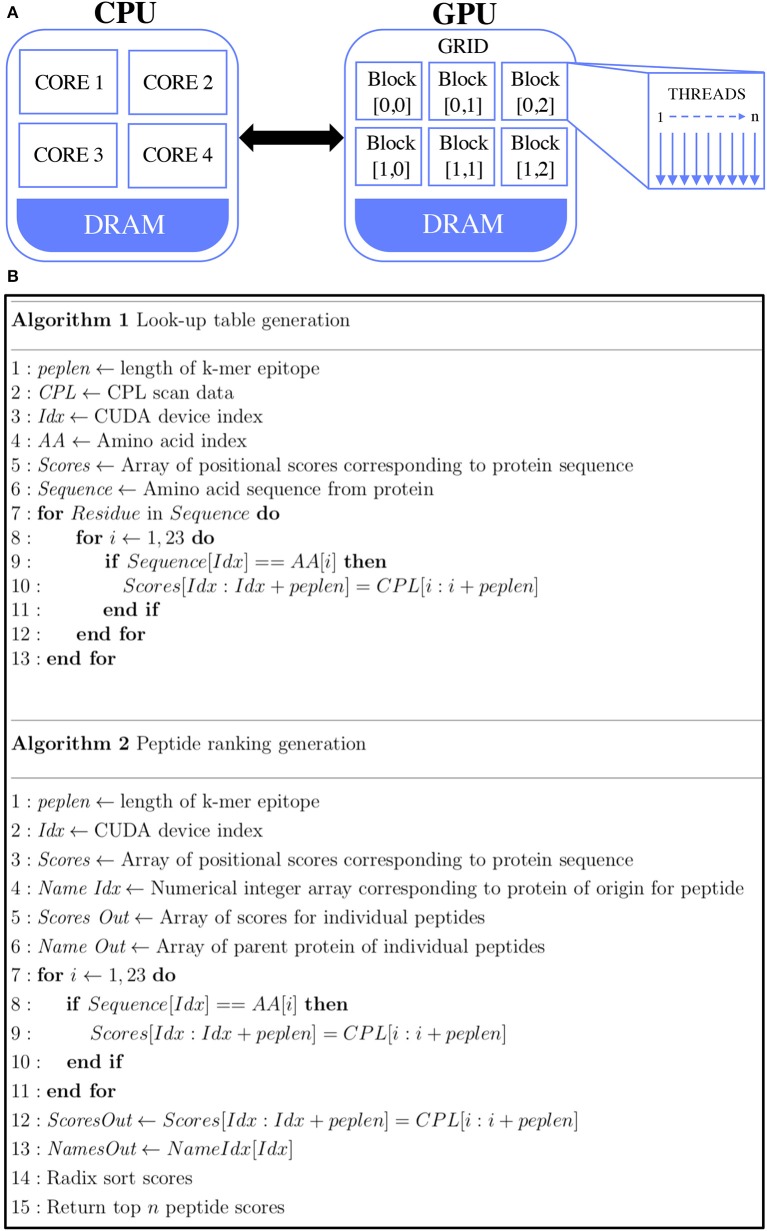
GPU-accelerated peptide scoring algorithm. **(A)** Differences in architecture differences between CPU (left) and GPU (right). Traditionally, computations are performed by transferring data from global memory to the CPU cores. GPU computing allows for the transfer of data from global memory to device memory, where the GPU cores can access them. **(B)** Pseudocode of the two main GPU parallel functions. Algorithm 1 (top) describes the look-up table generation of the peptides and algorithm 2 (bottom) describes the calculation of the agonist likelihood scores.

A more efficient approach to optimising the algorithm is to use GP-GPU programming, which takes advantage of GPU architecture that emphasises the parallel execution of very large numbers of relatively simple processes. The architecture differences between CPU and GPU are shown in Figure 1A. One operation is performed on many datasets (e.g., our protein databases are broken up into 30,000 protein sequences at a time and a single thread is launched for every protein sequence, thus analysing the data across multiple GPU blocks). Although Matlab provides a simple way to develop a prototype as the Parallel Computing Toolbox supports GP-GPU, substantial gains could be achieved by rewriting the program in C++. The code was optimised to minimise bottlenecks in serial processing and was adapted to run effectively on the GPU. This meant performing code refactoring to minimise memory allocation and transfer between the CPU and GPU by unrolling loops and passing every sequence together as a single one-dimensional array.

The look-up table and CPL algorithms are shown in Figure 1B. The CUDA code contains two main functions. The first function is the generation of the CPL scan data look-up table (Algorithm 1). In this, for every given amino acid a single thread was launched. This look-up table is kept in the GPU memory, where every k-mer is scored by its amino acid composition and positional score (Algorithm 2) which is done with one thread for every sequence. Here, the GPU RAM becomes a limiting factor. In the case of the Nvidia® Quadro K1200, this is 4GB of memory, which equates to about 30,000 sequences, along with the corresponding scoring data required. Once every potential agonist is scored in every given sequence, the scores are sorted in descending order based on their agonist likelihood score (Λ), a concept introduced in our previous study (17). The Λ-score makes use of the CPL scan data which essentially represents the recognition preference of a T-cell at each residue position, on average, over all its agonists. The peptide ranking is performed with the Thrust library of Bell and Hoberock (40). An in-built radix sort is used to sort the peptides, as this has been proven to be well-implemented in the CUDA code. An output file containing the score, peptide and parent sequence name are returned.

### Benchmarking

Benchmarking was performed on four different desktop implementations of the algorithm: a single core implementation, a 4 core OpenMP parallel, an 8 core OpenMP implementation and a CUDA implementation. The CUDA libraries plus compiler and OpenMP are freely downloadable from https://developer.nvidia.com/cuda-toolkit and https://www.openmp.org, respectively.

Two benchmarking tests were performed to investigate the execution time. The first test was undertaken by running each implementation of the code across databases of varying sizes; ranging from 10^6^ to 2 × 10^7^ sequences. For consistency, the database was an artificially generated set of protein sequences 311 amino acids in length (the average protein length found in the bacterial database). The number of results returned and the CPL data used were the same in each test. The second test varied the number of results returned (i.e., the number of top-scoring peptides), ranging from 100 to 10^6^ against a pathogenic fungal database consisting of 1.5 × 10^6^ sequences. All code was benchmarked on a machine running Ubuntu GNOME 16.10, with a Nvidia® Quadro K1200 graphics card with 512 cores, an Intel Core 6700-k processor and 16 GB of memory. All GP-GPU applications were developed using CUDA, hence were compiled with the nvcc compiler (v8.0.44). The sequential C++ code was compiled using the GNU g++ compiler (v5.4.0) with the C++11 ANSI standard and using the -O1/-O2/-O3 parameter option. OpenMP parallel code was compiled with the same GNU compiler, with an OpenMP 4.0 compiler directive.

### From Databases to Peptide Universe

The search space of the protein databases represents a tiny subsection of all possible peptides. For a given 9-mer, there are 20^9^ (5.1 × 10^11^) different unique amino acid combinations. This dwarfs the 16 billion peptides observed in the full bacterial universe. As well as the database-driven peptide-scoring algorithm, we have developed an optimised algorithm to score a selected peptide against the *entire* peptide universe for given a CPL data. The code once again leverages the CUDA library to optimise for speed. The *in silico* search utilises the GPU's architecture to parallelise in 3 dimensions. Therefore, every possible peptide is generated by means of collecting all sub-peptides a third of the total peptide size (e.g., for a 9-mer this would be 8,000 × 3 sets of 3-mers); which are then combined across all permutations on the GPU. The CUDA- and C++-version of the algorithm is available at https://github.com/WhalleyT/PICPL, within the downloadable executable.

## Results

### T-Cell Clone InsB4 Recognises a *bona fide* HLA A2-Restricted Insulin Epitope Presented at the Surface of Human β-Cells

**Figure 2:**
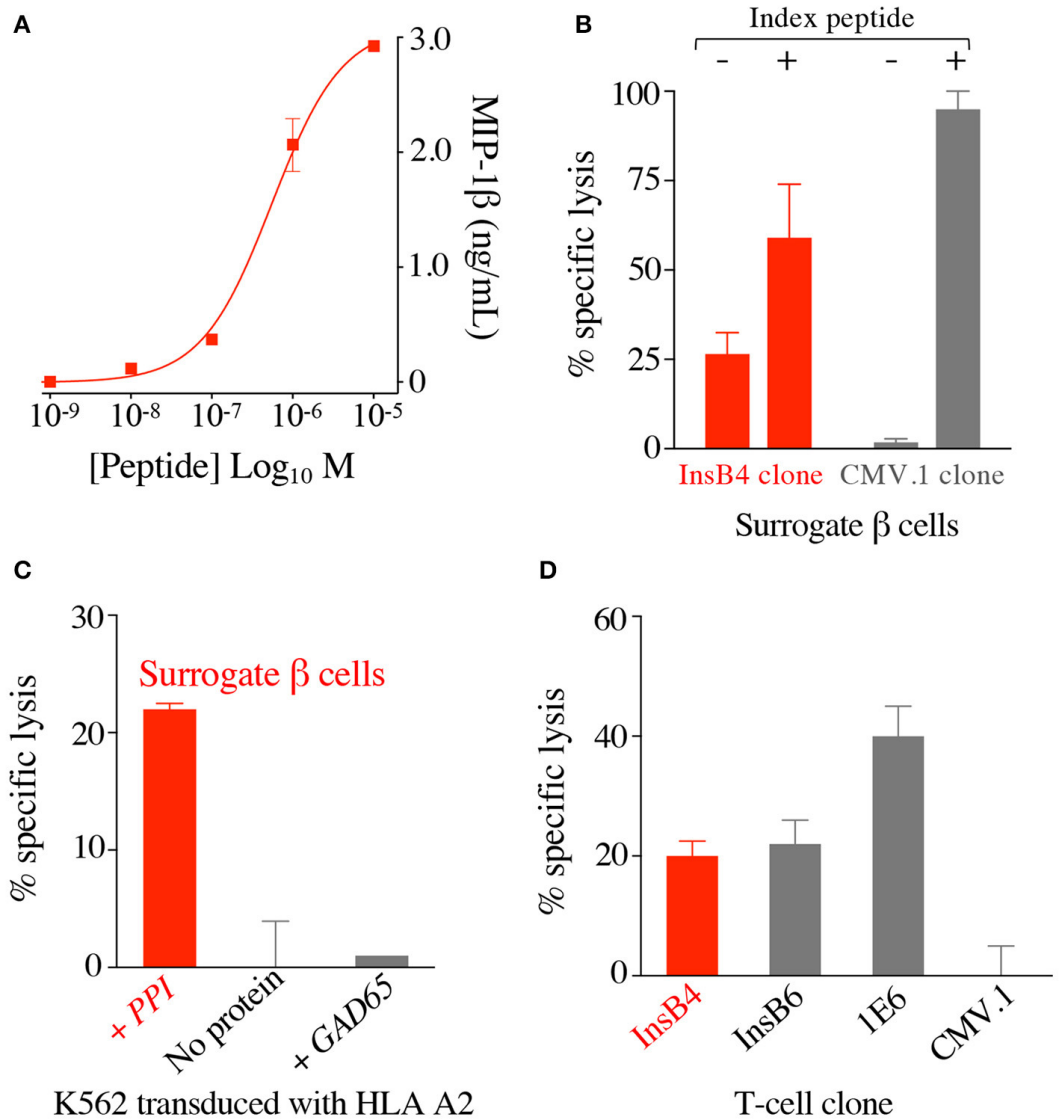
T-cell clone InsB4 recognises a genuine HLA A2-restricted, insulin B-derived epitope presented at the surface of human pancreatic β cells. **(A)** HLA A2 restricted clone InsB4 grown from a patient with type 1 diabetes recognizes the peptide HLVEALYLV from insulin β chain. InsB4 tested with an increasing concentration of the HLVEALYLV peptide according to the x-axis. Performed in duplicate: overnight incubation and MIP-1β ELISA. Error bars depict SEM. **(B)** Surrogate β-cells (K562 transduced with HLA A2 and preproinsulin) were killed by the InsB4 clone. Chromium release cytotoxicity assay at a T-cell to target cell ratio of 3:1 over 5 h. CMV.1 (pp65 residues 495–503 from CMV) clone used as an irrelevant control. Exogenous index peptides (10^−5^ M) for each clone were included as positive controls for killing of the surrogate β-cells. Error bars depict SEM. **(C)** InsB4 specifically killed surrogate pancreatic β-cells as K562s transduced with HLA A2 alone or GAD65 (irrelevant protein) and HLA A2 were not recognised. Chromium release cytotoxicity assay at a T-cell to target cell ratio of 3:1 over 5 h. Performed in duplicate with error bars depicting SEM. Assay performed twice with similar results. **(D)** InsB4 and sister clone InsB6 (expressing the same TCR) kills real pancreatic cells. HLA A2 restricted clones 1E6 (preproinsulin (PPI) residues 15–24) and CMV.1 (pp65 residues 495–503 from CMV) were used as positive and negative controls, respectively. Performed in duplicate with error bars depicting SEM.

T-cell clone GD.InsB4 (InsB4 from hereon) was generated from the blood of a patient with T1D using the T-cell libraries approach and the putative human insulin β chain_10−18_ epitope, HLVEALYLV, as previously described (26), with peptide activation data shown in Figure 2A. This clone was initially shown to recognise K562 cells transduced with HLA A2 and preproinsulin (so-called surrogate human β-cells), which were not killed by a T-cell clone with a different specificity (Figure 2B). Killing of the surrogate cells by InsB4 was dependent on expression of preproinsulin as K562 cells transduced HLA A2 alone or with HLA A2 and GAD65 were not recognised (Figure 2C). These results show that the HLVEALYLV epitope can be genuinely processed and presented at the cell surface in the context of HLA A2. We next demonstrated that InsB4, sister clone GD.InsB6 (InsB6) and preproinsulin-specific T-cell clone 1E6 (14) could lyse real human pancreatic β-cells (Figure 2D). A control clone, CMV.1 specific for the CMV pp65-derived epitope NLVPMVATV, did not kill these pancreatic cells in parallel assays. We conclude that InsB4 and InsB6 clones, derived from the blood of a patient with T1D can destroy human pancreatic β-cells and therefore have capacity to cause T1D. Importantly, these data represent the first time that the HLVEALYLV peptide has been confirmed as a genuine human β-cell epitope that is processed and presented by HLA A2^+^ pancreatic cells and capable of resulting in destruction of insulin producing cells.

### CPL Screening of T-Cell Clone InsB4

**Figure 3:**
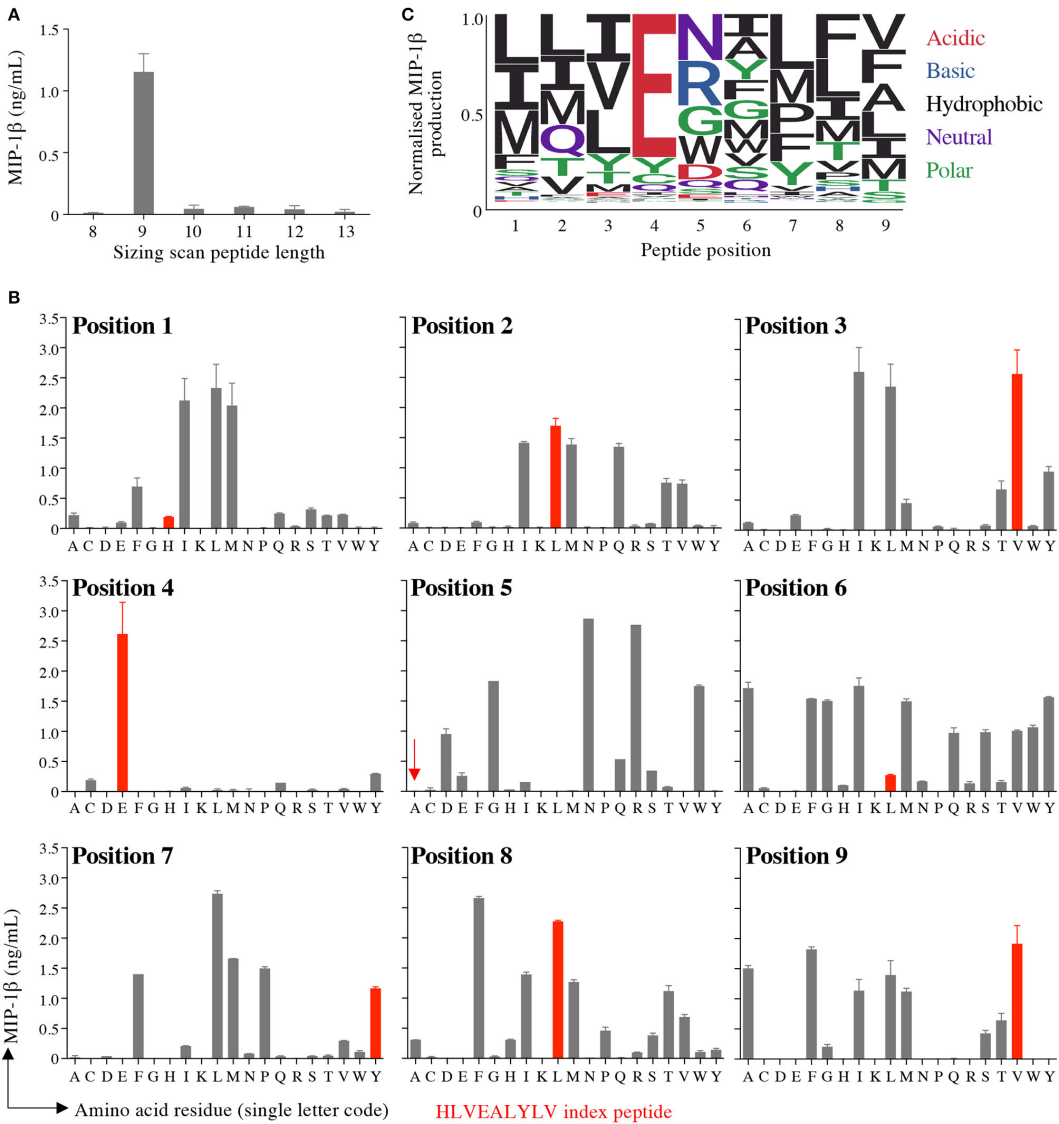
Sizing scan and nonamer combinatorial peptide library screening of InsB4. **(A)** Sizing scan of the InsB4 clone; overnight activation in duplicate followed by MIP-1β ELISA. Error bars depict SEM. **(B)** Overnight activation in duplicate with MIP-1β ELISA. Index peptide residues shown in red. Error bars depict SEM. **(C)** Logo plot of normalised MIP-1β expression from data presented in panel B.

Preference of InsB4 for nonamer peptides was confirmed by performing a sizing scan using random peptide mixtures of 8, 9, 10, 11, 12, and 13 amino acids in length (Figure 3A). This sizing scan indicated that the InsB4 T-cell exhibited a strong preference for peptides of 9 amino acids in length. We next undertook nonamer combinatorial library screening of the InsB4 clone. MIP1β amounts produced by each of the 180 sublibraries in the PS-CPL scan is shown in Figure 3B and summarised as a logo plot in Figure 3C. The “index” glutamic acid sub-library in position 4 was recognised most strongly, with virtually no evidence that strong agonists display any other amino acid residue at this position. The strong preference for a glutamic acid residue at position 4 suggests that this residue may form the major TCR contact and, moreover, that the InsB4 TCR might focus on a precisely delimited hotspot. Sub-libraries with index peptide residues (i.e., those residues found in the HLVEALYLV insulin-derived peptide) at positions 1, 5 and 6 were recognised poorly. Selected sub-libraries at positions 1, 5, 6, 7, and 8 produced more MIP1β than the index sub-library suggesting that it might be possible to create super-agonist peptides for this TCR that exhibit greater functional sensitivity than the index peptide sequence.

### Enhanced Screening of Curated Pathogen Databases Using GPUs

We assembled databases containing protein sequences from fungi and bacteria. We have previously collated a database of viral proteomes of species that infect, or might infect, humans (17). The bacterial proteome database was subdivided into two pathogenicity classes: category I (species not known to cause, or which have been proven *not* to cause, disease in humans or animals) and category II (species known to infect humans or animals, including species that can cause human disease). For a given CPL scan, evaluation of 1,573,341 distinct, proteins from fungal pathogens and 11,838,978 distinct category I and 11,589,864 distinct category II bacterial proteins took 2, 12, and 13 min, respectively, on the GPU (outputting the 2000 top-scoring peptides in each case). The links to the publicly available fungal and bacterial databases are provided in the Supplementary Material.

### GPU Computing Efficiency

**Figure 4:**
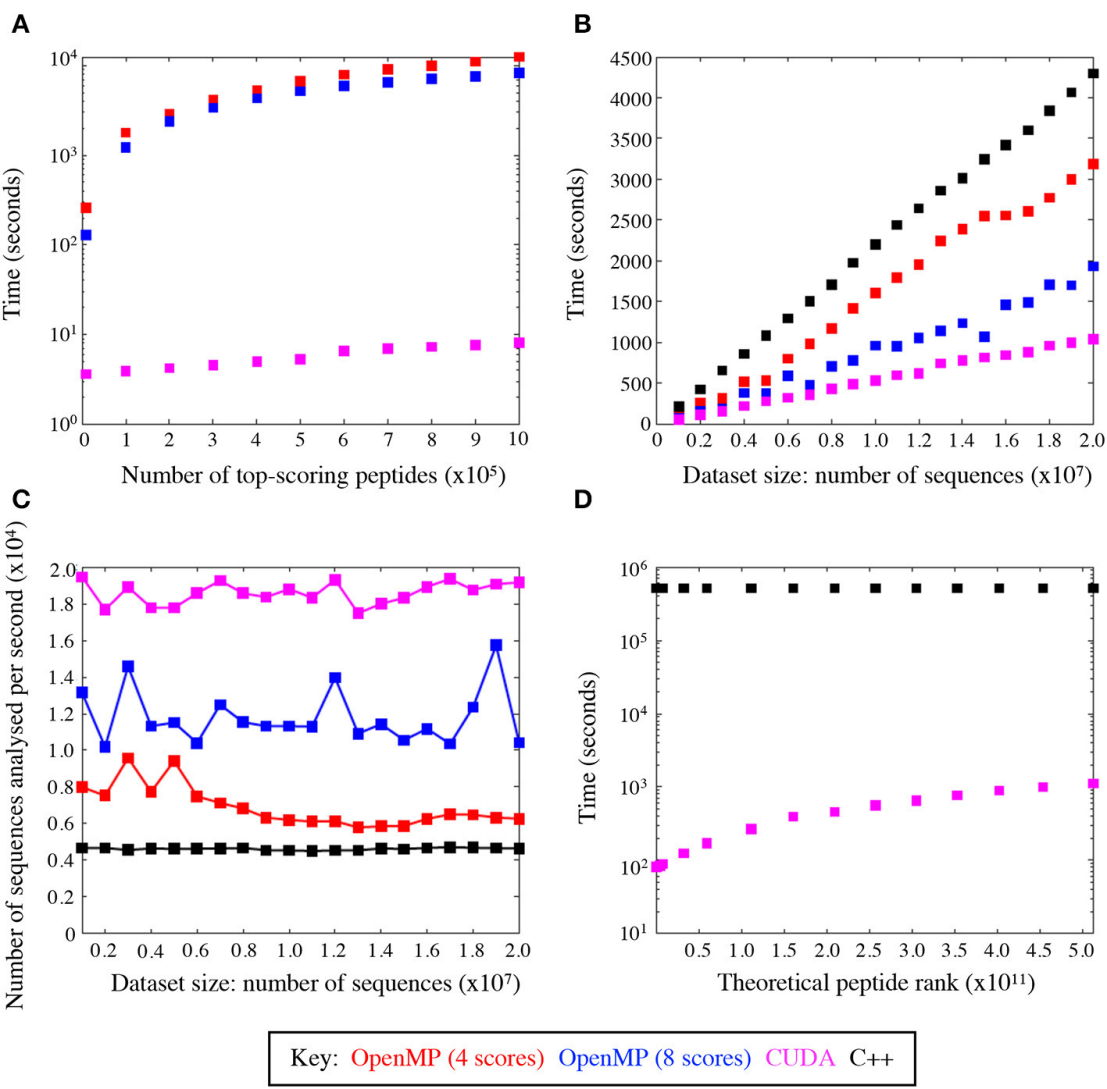
GPU speed-up compared with C++ and OpenMP. **(A)** Average total runtime of the peptide scoring algorithm, where the number of returned top-scoring peptides varied from 100 to 10^6^. **(B)** Average speed-up across C++ and OpenMP implementations, where the size of the dataset to scan varied from 10^6^ to 2 × 10^7^ sequences. **(C)** The average rate at which sequences were analysed with CUDA is superior to all the other implementations. **(D)** Average speed-up by the CUDA and C++ code to rank theoretical peptides ordered from the highest to the lowest agonist likelihood score.

We evaluated the training performance of GPU acceleration on the number of top-ranked peptides and datasets of varying sizes and compared its performance with other platforms. When compared with the number of top-ranked peptides, the CUDA implementation of the algorithm was seemingly unaffected by the number of top-scoring peptides, unlike the other platforms (Figure 4A). GPU was superior when tested on datasets of different sizes and reduced the time to scan datasets by a factor of ~4.5 compared to C++ (Figure 4B) thus giving a much broader search depth. Figure 4C shows the average speed-up for datasets of different sizes. The CUDA implementation analysed an average of 18,589.4 sequences per second compared to 4,624.2 sequences and 11,800.6 sequences per second in the serial (C++) and OpenMP implementations, respectively. This results in a marked reduction of run time, which is conserved across varying input data sizes.

The CUDA code was also profiled using the Nvidia® profiler (https://developer.nvidia.com/nvidiavisual-profiler) which demonstrated that the GPU parallelisation removed the scoring function as the main bottleneck, leaving input/output operations as the limiting factor on the run time. This was evident from the Nvidia® profiler output, which attributed input/output operations (i.e., reading and writing the files to and from disk) to take up 28% of the code's run time. The functional CUDA code took 11% of the time with the rest being attributed to CPU dependent operations. This shows that even with substantial further optimisation, the main time losses would be incurred with operations that are not easily relegated to the GPU.

Figure 4D compares the speed-up between the GPU and C++ codes scanned against the entire peptide universe. When comparing the two extreme cases for the highest (LLIENILFV) and the lowest (GGCAADCDC) scoring possible peptides, we observed a significant increase in time. This is because every time the queried peptide scores lower than a given peptide the GPU threads must be synchronised and halted while memory is accessed to add to the ranking value. The highest scoring peptide was ranked in 82 s, whereas the lowest scoring peptide was ranked in 1,117 s leading to >10-fold increase in runtime. Nevertheless, the GPU-accelerated algorithm performed significantly better than its serial C++ implementation, which took 16,088 and 16,144 s for the best and worst case, respectively. The index insulin-derived peptide (HLVEALYLV) ranked at 789,930,969 (still within the top 0.15% of all nonamers) in 85 s.

### Validation of the GPU-Accelerated Scoring Algorithm With Randomly Chosen Uniformly Distributed Pathogen-Derived Peptides

We tested the GPU-accelerated strategy by interrogating the fungal and bacterial pathoproteome for peptides predicted to activate the autoimmune InsB4 clone. The PS-CPL data depicted in Figure 3 were used to screen the protein databases compiled from fungal or bacterial species, as described above. As part of the database screening, a searching algorithm generates a hierarchal list of possible agonists by assigning each peptide with an agonist likelihood score (Λ) developed in (17). We previously demonstrated that the cognate peptide for antiviral or antitumor clones could be identified using PS-CPL data, pathogenic viral (10,733 proteins) or human self (54,886 proteins) databases and the scoring algorithm using CPU, with a strong association between the Λ-value, and the peptide's functional sensitivity to activate the respective clone (17). Here, our objective was to establish if the GPU-accelerated algorithm could identify potential agonists from the pathogenic fungi database (1,573,341 proteins) and the entire pathogenic bacterial database (23,428,842 proteins).

For the purpose of validation, 72 fungal and 72 bacterial peptides were chosen, which were uniformly sampled without repetition and whose Λ-values spanned ~6 orders of magnitude, thus placing 12 peptides in six “bins,” with each bin representing an order of magnitude. To achieve a sufficiently large span of unique Λ-values for sampling, the top 3 × 10^5^ fungal and top 5 × 10^6^ bacterial peptides were ranked by the searching algorithm based on the CPL data. This was only feasible by using multiple cores on GPUs, which increased the speed of peptide identification from >1 day to under 3 and 11 min, for the fungal and bacterial sequences, respectively.

**Figure 5:**
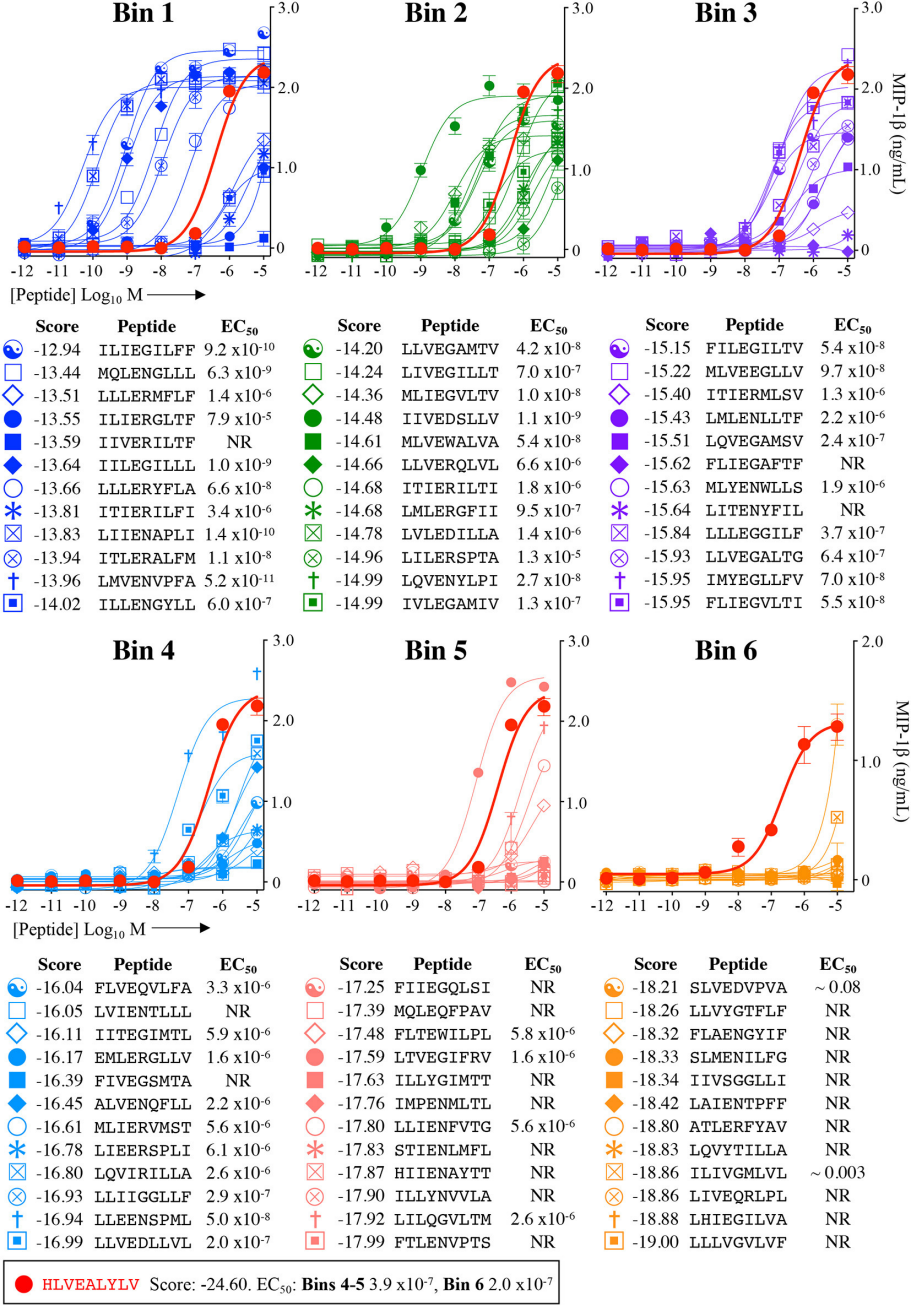
Recognition of 72 randomly chosen and uniformly distributed fungal peptides by the InsB4 T-cell clone. Each panel shows results of MIP-1β assay, indicating activation of T-cell clone InsB4 by 12 peptides and the index HLVEALYLV peptide (shown as a bold red line). InsB4 was incubated overnight with the indicated peptides and MIP-1β released into the supernatants quantified by ELISA. Bins 1–5 were performed in the same assay with Bin 6 performed in a separate experiment hence the different EC_50_ values for the index peptide. EC_50_ for each peptide (M) is displayed in the tables. NR, no T-cell response. The mean *p*EC_50_ for each bin is plotted in Figure 7A.

**Figure 6:**
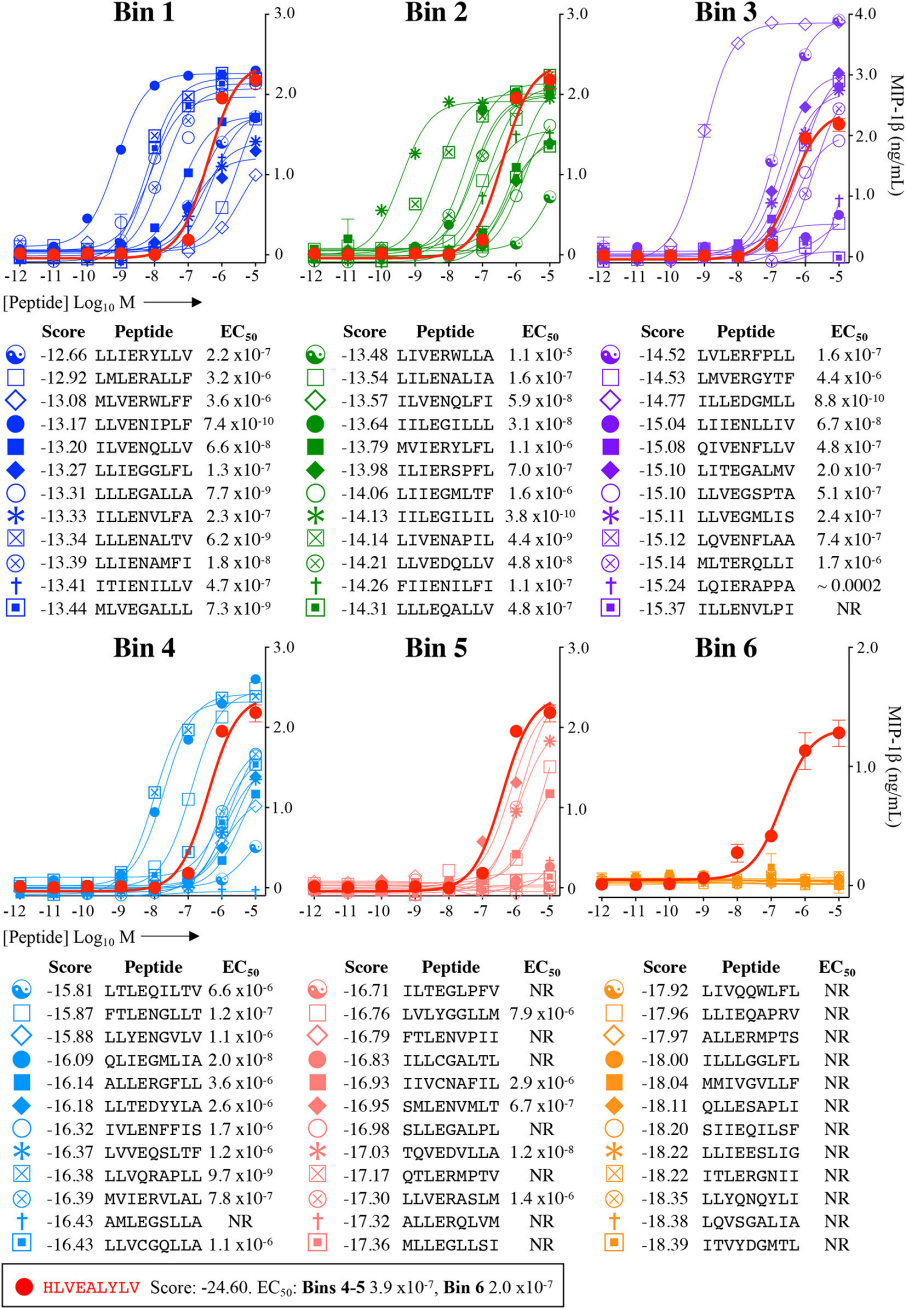
Recognition of 72 randomly chosen and uniformly distributed bacterial peptides by the InsB4 T-cell clone. Each panel shows results of MIP-1β assay, indicating activation of T-cell clone InsB4 by 12 peptides and the index HLVEALYLV peptide (shown as a bold red line). InsB4 was incubated overnight with the indicated peptides and MIP-1β released into the supernatants quantified by ELISA. Bins 1-5 were performed in the same assay with Bin 6 performed in a separate experiment hence the different EC_50_ values for the index peptide. EC_50_ for each peptide (M) is displayed in the tables. NR, no T-cell response. The mean *p*EC_50_ for each bin is plotted in Figure 7A.

**Figure 7:**
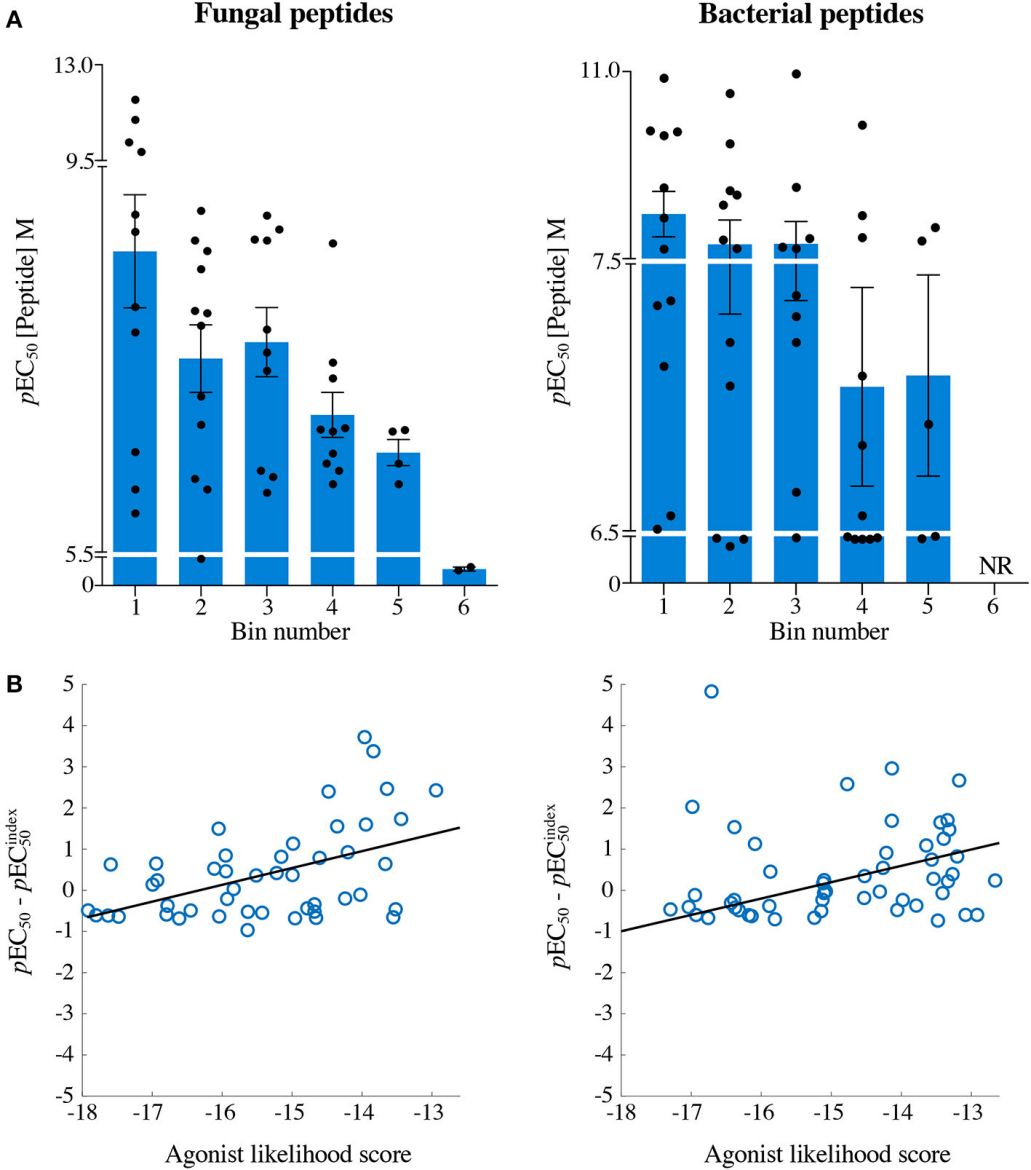
The agonist likelihood score is able to identify fungal and bacterial peptide agonists. **(A)** *p*EC_50_ values of InsB4 clone activation towards randomly sampled peptides shown in Figures 5, 6. NR, no T-cell response. Solid bars depict the mean and error bars the SEM. **(B)** Scatter plot of relative functional sensitivity (Δ*p*EC_50_) for the 72 fungal and 72 bacterial randomly sampled peptides shown in Figures 5, 6 vs. agonist likelihood score.

Each peptide was tested for functional sensitivity in titration assays, with InsB4 activation quantified by MIP-1β ELISA (Figures 5, 6). For the fungal protein-derived peptides it was evident that the most potent agonists resided in Bin 1 with 7 sequences being more potent than the index insulin_10−18_ sequence (Figure 5). Average ligand functional sensitivity decreased through Bins 2–5 such that activation was only observed with 4/12 peptides in the 5th Bin. Results were similar for peptides identified from the much larger bacterial protein database (Figures 6, 7A) except the greater number of overall peptides resulted in more being found that were of higher functional sensitivity than the index insulin_10−18_ sequence. The most potent sequences found were LLVENIPLF (Bin 1), IILEGILIL from (Bin 2), and ILLEDGMLL (Bin 3). None of the peptides in Bin 6 acted as agonists of the InsB4 T-cell (Figures 6, 7A).

Overall, it was apparent that peptides that scored as being most likely to be seen by InsB4 were indeed better agonists for InsB4, with fewer peptides being recognised when moving from Bin 1 to Bin 6, for both the fungal and bacterial peptides (Figure 7A). In order to make comprehensive comparisons between the 144 peptides, the *p*EC_50_ (defined as –log_10_ EC_50_) of InsB4 activation for every peptide was compared to that of the HLVEALYLV index $\left(p\text{EC}\begin{array}{c} index\\ 50 \end{array}\right)$, giving each peptide a relative functional sensitivity (Δ*p*EC_50_ = *p*EC_50_ – $p\text{EC}\begin{array}{c} index\\ 50 \end{array}$). We examined the statistical association between the agonist likelihood score Λ and the relative functional sensitivity Δ*p*EC_50_ for all 144 randomly chosen peptides using both the Spearman's and Pearson's correlation coefficient (Figure 7B). For the fungal peptides, Spearman correlation was 0.42 (significant at the 0.5% level) and the Pearson correlation was 0.48 (significant at the 0.1% level). For the bacterial peptides, Spearman correlation was 0.36 (significant at the 0.1% level) and the Pearson correlation was 0.39 (significant at the 0.1% level). These results demonstrate that the CPL-driven database searching can accurately identify peptides derived from cellular organisms that were recognised by an autoimmune T-cell clone.

### Pathogen-Derived Peptides Can Act as Super-Agonist Ligands for an Insulin B-Specific T-Cell Clone

**Figure 8:**
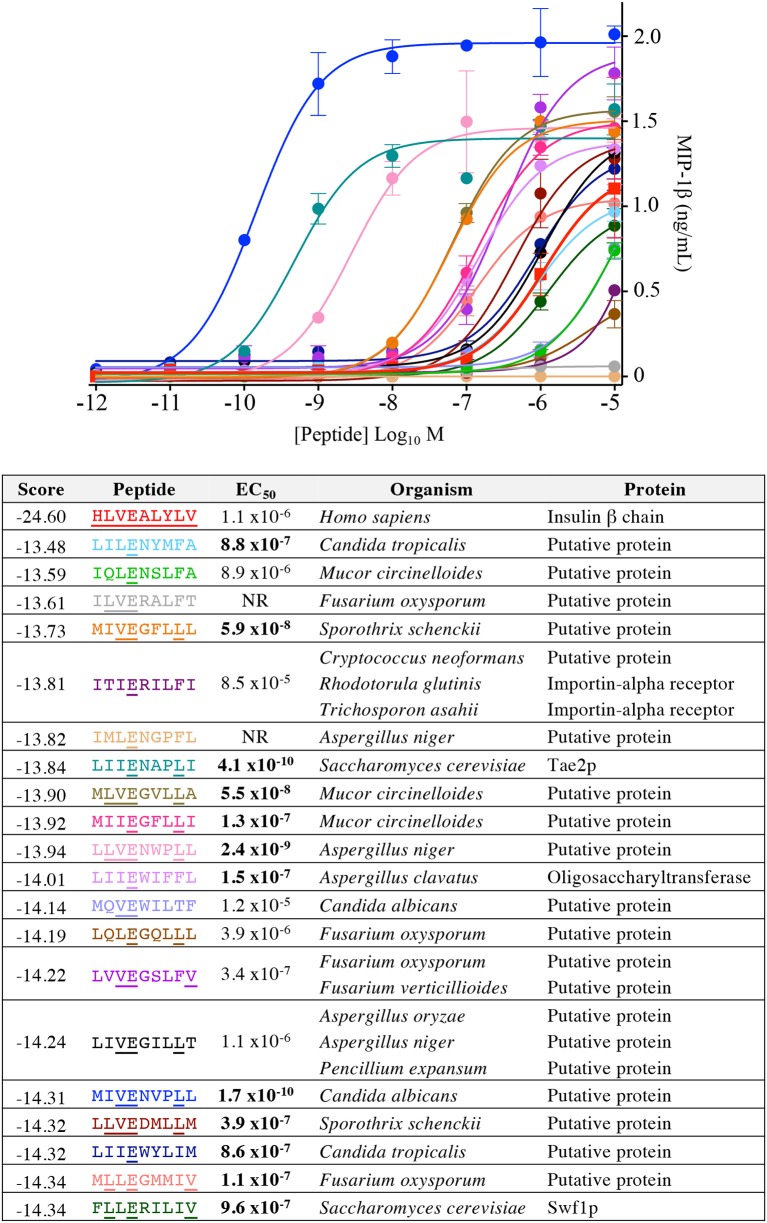
InsB4 recognizes fungal peptides with increased sensitivity relative to index insulin peptide. The 20 top-scoring peptides from fungal species were tested overnight for activation on InsB4 by MIP-1β ELISA. The likelihood scores and EC_50_ of peptide (M) activation are shown. Putative proteins are the product of predicted open reading frames from the genome sequence of the species indicated. NR, no T-cell response. Bold EC_50_ more potent than the index peptide. Amino acid residues that are the same for each fungal peptide and the insulin index peptide (HLVEALYLV) are underlined.

**Figure 9:**
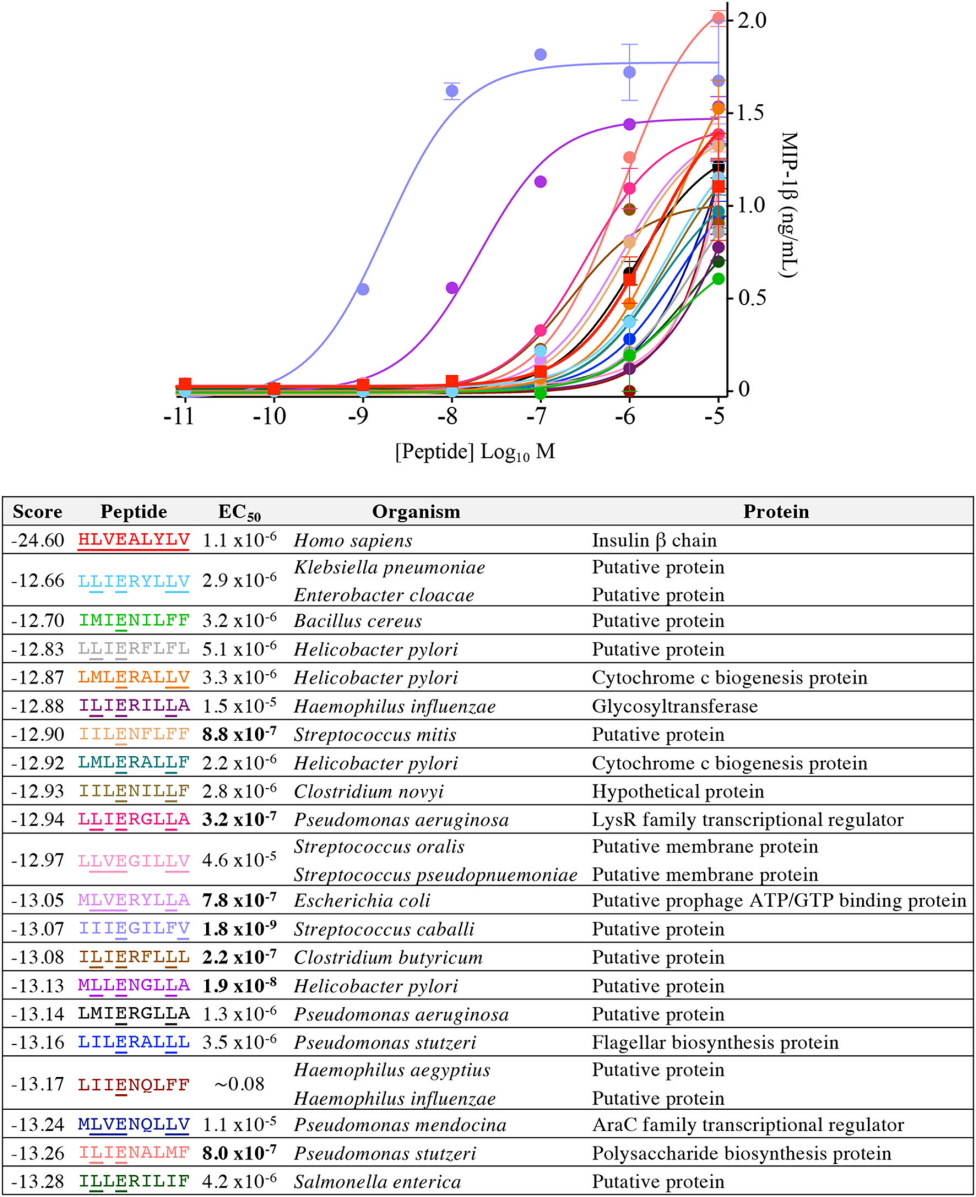
InsB4 recognizes bacterial peptides with increased sensitivity relative to index insulin peptide. The 20 top-scoring peptides from category II (i.e., confirmed pathogenic) bacterial species were tested overnight for activation on InsB4 by MIP-1β ELISA. The likelihood scores and EC_50_ of peptide (M) activation are shown. Putative proteins are the product of predicted open reading frames from the genome sequence of the species indicated. Bold EC_50_ more potent than the index peptide. ~ = ambiguous EC_50_ based on GraphPad Prism software analysis. Amino acid residues that are the same for each bacterial peptide and the insulin index peptide (HLVEALYLV) are underlined.

Our CPL-based peptide searching algorithm identified several peptides derived from bacterial and fungal proteins that shared a very low sequence homology with the index insulin-derived peptide but were recognised by the InsB4 T-cell clone with higher functional sensitivity. Whereas, the above approach randomly sampled peptides from the bacterial and fungal databases, we next explored the top scoring peptides from each database and functionally tested 20 peptides. As shown in Figure 8, the top 5 most potent fungal InsB4 ligands were peptides derived from *Candida albicans* (MIVENVPLL), *Saccharomyces cerevisiae* (LIIENAPLI), *Aspergillus niger* (LLVENWPLL), *Mucor circinelloides* (MLVEGVLLA), and *Sporothrix schenckii* (MIVEGFLLL). As shown in Figure 9, all 20 bacterial peptides for InsB4 were recognised (19 gave unambiguous EC_50_ values), with 7 of the peptides eliciting greater activation from InsB4 than the index HLVEALYLV insulin-derived peptide. Furthermore, peptides from *Streptococcus caballi* (IIIEGILFV) and *Helicobacter pylori* (MLLENGLLA) acted as super agonists of InsB4. These data strongly suggest that the InsB4 TCR can bind to pathogen-derived peptides with higher affinity than with the index HLVEALYLV sequence that can mediate destruction of human pancreatic β-cells.

**Figure 10:**
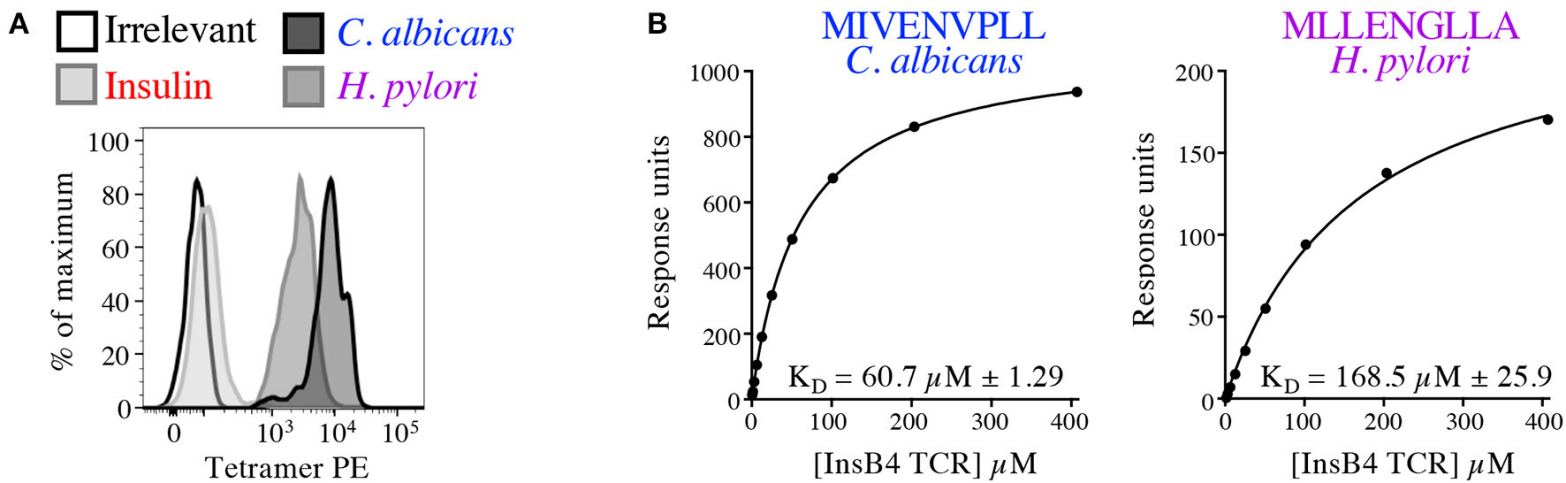
Pathogen-derived peptide ligands bind to the InsB4 TCR. **(A)** Optimised pMHC tetramer staining of the InsB4 T-cell clone with HLA A2-HLVEALYLV (insulin), HLA A2-MLLENGLLA (*H. pylori*) and HLA A2-MIVENVPLL (*C. albicans*) epitopes. **(B)** SPR binding data of the interaction between the InsB4 TCR and HLA A2-MIVENVPLL and HLA A2-MLLENGLLA. Binding with the index HLA A2-HLVEALYLV peptide was too weak to measure.

In order to formally prove enhanced TCR binding we examined pMHC tetramer staining and ligand interaction with soluble InsB4 TCR by SPR. The InsB4 T-cell clone stained well with tetramers made with HLA A2-MIVENVPLL (*C. albicans*) and HLA A2-MLLENGLLA (*H. pylori*) (Figure 10A). In contrast staining with the index peptide, insulin-derived HLA A2-HLVEALYLV tetramer was very weak. Peptide-HLA tetramer staining intensity is known to correlate with TCR affinity for the cognate ligand (29). Autoimmune TCRs are known to stain poorly with cognate tetramer (35, 41), so this weak staining was not unexpected. Interaction between the InsB4 TCR and HLA A2-HLVEALYLV was too weak to measure by SPR. In contrast, *C. albicans*-derived HLA A2-MIVENVPLL and *H. pylori*-derived HLA A2-MLLENGLLA antigens bound with a K_D_ of 60.7 and 168.5 μM, respectively, confirming that these peptides engage the TCR within an affinity range capable of triggering T-cell activation (Figure 10B). Attempts to gather structural data of the InsB4 TCR in complex with a cognate antigen failed as we were unable to grow crystals of this TCR, despite multiple attempts.

## Discussion

There is a pressing need for efficient computational methodology for prediction of peptide mimics and super-agonists for therapeutically relevant T-cell clones. Here, we first validated a human insulin β chain_10−18_ epitope HLVEALYLV using T1D patient-derived T-cell clone InsB4 and showed that this T-cell could kill human pancreatic β-cells. These data represent the first time that the HLVEALYLV peptide has been confirmed as a naturally processed β-cell-presented epitope. Recognition patterns of random peptide mixtures of 8, 9, 10, 11, 12, and 13 amino acids in length clearly showed that the InsB4 T-cell preferred nonamer peptides. In order to investigate the peptide degeneracy profile of InsB4 we performed a nonamer PS-CPL screen. InsB4 demonstrated a clear preference for a glutamic acid residue at position 4 strongly suggesting that this residue acts as the primary TCR contact within the peptide. In support of this notion, this residue was prominent in all the super-agonist peptides we extracted from the pathoproteome.

In order to determine whether the InsB4 T-cell could cross-react with pathogen-derived peptides we turned to computational screening of PS-CPL data. *In silico* searching of the known terrestrial proteome with our algorithm previously developed using Matlab (17) takes several days on an HPC server. We reduced the time for these searches to under 30 min using GP-GPU allowing this approach to be run on a desktop or laptop computer. The search algorithm generates a list of potential agonists by assigning to each peptide an agonist likelihood score (17). Testing with 144 peptides selected uniformly across a logarithmic scale of the lambda score from the fungal and bacterial pathoproteome with values spanning ~6 orders of magnitude demonstrated that the CPL-driven database searching could accurately identify peptides derived from cellular organisms that were recognised by an autoimmune T-cell clone. Functional testing of 20 high scoring peptides from the new bacterial and fungal databases showed that 20/20 and 18/20 acted as agonists of the InsB4 insulin-specific T-cell clone. Furthermore, 7/20 and 12/20 sequences predicted from the bacterial and fungal databases were more potent agonists than the HLVEALYLV index sequence. In theory, if presented *in vivo*, such super-agonist peptides might have the capacity to break tolerance to trigger disease. Importantly, one bacterial and one fungal peptide derived from *H. pylori* and *C. albicans*, respectively, were selected to demonstrate conclusively that they could engage the InsB4 autoimmune TCR as peptide-MHC tetramers and by SPR. Interestingly, several recent studies have shown an increased rate of *H. pylori* infection in T1D patients (42–45) leading to suggestions that it may be a disease trigger; however, it is also possible that T1D might render patients more susceptible to *H. pylori* infection. Similarly, T1D has also been linked with *C. albicans* at the time of diagnosis (46) but diabetes is known to predispose individuals to fungal infections (47) so the direction of any link is still in question.

The resemblance between epitopes derived from microbial and host proteins leading to crossreactivity of T-cells in the host has been termed molecular mimicry (48, 49) and is believed to be a major mechanism by which autoimmune diseases are triggered (50, 51). Indeed, T-cell crossreactivity between microbially-derived peptides and self-peptides can induce autoimmunity in experimental animal models (52–54). Recent studies have confirmed that TCRs are capable of recognising large numbers of different individual peptides in the context of a single MHC molecule (7, 55, 56). Several mechanisms for TCR promiscuity have been suggested that include; macrolevel changes in peptide binding register, alterations in TCR crossing angle on peptide-MHC (pMHC), TCR CDR loop flexibility, and residue focused “hotspot” binding (1). Evidence for these mechanisms is emerging and points to further unanticipated molecular flexibility that can extend the range of peptides recognised via individual TCRs. The TCR can interact with different pMHCs using a common (55, 57–60), or a highly divergent binding mode (61). The structure of TCR CDR loops are known to differ in ligated and unligated TCR crystal structures suggesting that these loops are flexible and might be capable of binding to different peptides in different ways (59, 62). However, it is worth noting that a recent study found large conformational differences of up to 5 Å in individual CDR loops in different crystals of the same unligated TCR, suggesting that crystal structures may only offer an artefactual indication of TCR flexibility due to lattice packing and crystallisation conditions (63). Two recent studies have described extensive peptide crossreactivity as the result of constrained interactions with peptide hotspots in MHC class I and class II-restricted peptides (16, 55). More recent studies have shown that heretofore unanticipated rearrangements in the peptide and presenting MHC protein can extend the range of ligands that can be recognised (64, 65). The potential consequences of widespread T-cell crossreactivity are profound and range from heterologous immunity, where an individual T-cell clone can target more than one pathogen via a single TCR, to crossreactivity between pathogen-derived and self-peptides [reviewed in (1)].

While the combined computational-experimental approach presented in this study can be used to discover potential infectious triggers for autoimmune disease. Further work will be required to reveal if any of the potential infection-derived peptides found by our work are genuinely presented to T-cells *in vivo*. Moreover, this combined computational-experimental approach also has the potential to be used to discover potent peptide agonists of any T-cell clone for use in TCR-optimised peptide skewing of the repertoire of T-cells (TOPSORT) (66) and to rank the agonist potential of D-amino acid peptides (67). The GPU-accelerated strategy has not only surpassed the previous framework (17) in terms of speed, cost-, and power-efficiency, but it has also demonstrated its robustness by addressing the theoretical peptide ranking problem. In sum, GPU-based computing is practical and within reach of users with a desk-top computer; as such, we expect that it will find multiple applications in other computationally demanding areas of immunology such as immune phenotyping and antigen receptor analysis.

## Data Availability Statement

All datasets generated for this study are included in the article/Supplementary Files.

## Author Contributions

TW, GD, PB, AW, AF, LW, JH, MA, HB, MC, RK, DC, MP, AS, and BS performed and/or directed experiments, analysed data, and/or critiqued the manuscript. AS, LW, and BS conceived, funded, and directed the project. AS, TW, GD, and BS wrote the manuscript.

### Conflict of Interest

The authors declare that the research was conducted in the absence of any commercial or financial relationships that could be construed as a potential conflict of interest.
