# Supplementary material for: GPU-Accelerated Discovery of Pathogen-Derived Molecular Mimics of a T-Cell Insulin Epitope
Source: Front Immunol. 2020 Feb 28;11:296. doi: 10.3389/fimmu.2020.00296 (PMC7058665; doi:10.3389/fimmu.2020.00296)
Supplement: Supplementary file 1 [file Data_Sheet_1.docx]

GPU-Accelerated Discovery of Pathogen-Derived Molecular Mimics

of a T-cell Insulin Epitope

**Thomas Whalley^1,2*^, Garry Dolton^1*^, Paul E. Brown^3^, Aaron Wall^1^, Linda Wooldridge^4^, Hugo van den Berg^5^, Anna Fuller^1^, Jade Hopkins^1^, Michael D. Crowther^1,6^, Meriem Attaf^1^, Robin R. Knight^7^, David K. Cole^1,8^, Mark Peakman^7^, Andrew K. Sewell^1,2^ and Barbara Szomolay^1,2^**

Correspondence: Professor Andrew Sewell, E-mail: sewellak@cardiff.ac.uk. Tel: +442920 687055

Supplementary Material

**Bacteria/Fungi data**

**Bacteria downloaded from:**

<ftp://ftp.ncbi.nih.gov/genomes/genbank/bacteria/> (latest assembly version, if not present then all assembly version)

<ftp://ftp.ncbi.nih.gov/genomes/refseq/bacteria/> (same as above)

<ftp://ftp.ncbi.nih.gov/refseq/release/bacteria/>

<http://www.uniprot.org/uniprot/> (taxonomy: bacteria, reviewed: yes (Swiss-Prot))

<http://www.pdb.org/pdb/home/home.do> (search, taxonomy: bacteria only, polymer type: sequence, reports: customizable table: sequence, macromolecular name, source)

**Fungi downloaded from:**

<ftp://ftp.ncbi.nih.gov/genomes/genbank/fungi/> (latest assembly version, if not present then all assembly version)

<ftp://ftp.ncbi.nih.gov/genomes/refseq/fungi/> (same as above)

<ftp://ftp.ncbi.nih.gov/refseq/release/fungi/>

<http://www.uniprot.org/uniprot/> (taxonomy: fungi, reviewed: yes (Swiss-Prot))

<http://www.pdb.org/pdb/home/home.do> (search, organism: fungi, polymer type: sequence, reports: customizable table: sequence, macromolecular name, source)

**Bacterial pathogen list collated from following sources:**

Classification of Bacterial Pathogens by Alex van Belkum, COGEM research report

Risk factors for human disease emergence, Taylor, Latham, Woolhouse, Phil. Trans. R. Soc. Lond. B356: 983-989, 2001.

Email communication with NCBI (document available upon request)

**Fungal pathogen list collated from following sources:**

Risk factors for human disease emergence, Taylor, Latham, Woolhouse, Phil. Trans. R. Soc. Lond.B356: 983-989, 2001.
